# Water quality and dissolved load in the Chirchik and Akhangaran river basins (Uzbekistan, Central Asia)

**DOI:** 10.1007/s10661-024-13014-1

**Published:** 2024-08-28

**Authors:** Silvia Fornasaro, Lisa Ghezzi, Nosir Shukurov, Maxim Petrov, Riccardo Petrini

**Affiliations:** 1 Dipartimento Di Scienze Della Terra, Università Di Pisa, Via Santa Maria 53, Pisa, 56126 Italy; 2 Institute of Geology and Geophysics, University of Geological Sciences, Olimlar Street 64, Tashkent, 100164 Uzbekistan

**Keywords:** Uzbekistan, Chirchik–Akhangaran basin, Solute sources, Inorganic pollutants, Water quality

## Abstract

Uzbekistan (Central Asia) is experiencing serious water stress as a consequence of altered climate regime, past over-exploitation, and dependence from neighboring countries for water supply. The Chirchik–Akhangaran drainage basin, in the Tashkent province of Uzbekistan, includes watersheds from the Middle Tien Shan Mountains escarpments and the downstream floodplain of the Chirchik and Akhangaran rivers, major tributaries of the Syrdarya river. Water in the Chirchik–Akhangaran basin is facing potential anthropogenic pressure from different sources at the scale of river reaches, from both industrial and agricultural activities. In this study, the major and trace element chemistry of surface water and groundwater from the Chirchik–Akhangaran basin were investigated, with the aim of addressing the geogenic and anthropogenic contributions to the dissolved load. The results indicate that the geochemistry of water from the upstream catchments reflects the weathering of exposed lithologies. A significant increase in Na^+^, K^+^, SO_4_^2−^, Cl^−^, and NO_3_^−^ was observed downstream, indicating loadings from fertilizers used in croplands. However, quality parameters suggest that waters are generally suitable for irrigation purposes, even if the total dissolved solid indicates a possible salinity hazard. The concentration of trace elements (including potentially toxic elements) was lower than the thresholds set for water quality by different regulations. However, an exceedingly high concentration of Zn, Mo, Sb, Pb, Ni, U, As, and B compared with the average river water worldwide was observed. Water in a coal fly-ash large pond related to the Angren coal-fired power plants stands out for the high As, Al, B, Mo, and Sb concentration, having a groundwater contamination potential during infiltration. Spring waters used for drinking purposes meet the World Health Organization and the Republic of Uzbekistan quality standards. However, a surveillance of such drinking-water supplies is suggested. The obtained results are indicators for an improved water resource management.

## Introduction

Water shortage and pollution due to climate change and anthropogenic activities are of major primary concern worldwide, threatening human health and sustainable development in many Countries (Salehi, 2022). In particular, changes in water availability and quality, consequences of altered climate regimes, represent a high-impact risk for the arid and semi-arid regions of Central Asia, where lowlands are characterized by a low amount of precipitation and reduced river flows (Bekturganov et al., 2016; Karthe et al., 2017; Wang et al., 2022). One of the dramatic effects of the water crisis is the rapid shrinking of the Aral Sea Basin due to the over-exploitation of its influents. In Central Asia, mountain ranges (Tien Shan, Pamir, and Hindu Kush) represent the primary water source for transboundary river basins (Karthe et al., 2017 and references therein). In these settings, a significant loss of the glacier mass and permafrost decline are observed (Bolch, 2007; Duishonakunov et al., 2014; Hagg et al., 2007; Hoelzle et al., 2019; Huss & Hock, 2018; Sorg et al., 2012), with long-term impacts on water resources in the distal and populated areas (Ajami et al., 2012; Didovets et al., 2021; Lutz et al., 2013; Markovich et al., 2019; Schreiner-McGraw & Ajami, 2022; Yapiyev et al., 2021).

Among Central Asia countries, Uzbekistan has the largest dependency on external flow from the upstream water-rich countries Tajikistan and Kyrgyzstan (Russell 2018; Didovets et al., 2021), still raising conflictive events (Chatalova et al., 2017; Groll et al., 2015; Wang et al., 2021). Indeed, Uzbekistan is characterized by severe water stress conditions (Karthe et al., 2017; Tian & Zhang, 2020; Wang et al., 2022), which are expected to worsen in the future (Duishonakunov et al., 2014; Shahgedanova et al., 2018). The Chirchik–Akhangaran river basin (CAB), in the northern Tashkent province of Uzbekistan, provides essential water supplies for agriculture, industrial activities, and domestic uses (Gafforov et al., 2023). In addition, Chirchik and Akhangaran contribute significantly to the Syrdarya river, a main tributary of the Aral Sea. In the CAB, water is mainly supplied from glaciers and snowmelt from the north-western segment of the Tien-Shan Mountain range (Bel’tau-Kurama range; Gafforov et al., 2023) flowing downstream through extensively anthropized areas (e.g., Makhmudova & Buriev, 2020).

In the present study, surface water and groundwater were collected in the CAB in order to investigate the geogenic and anthropogenic control of the dissolved load, with implications for water management plans.

## Study area

**Fig. 1:**
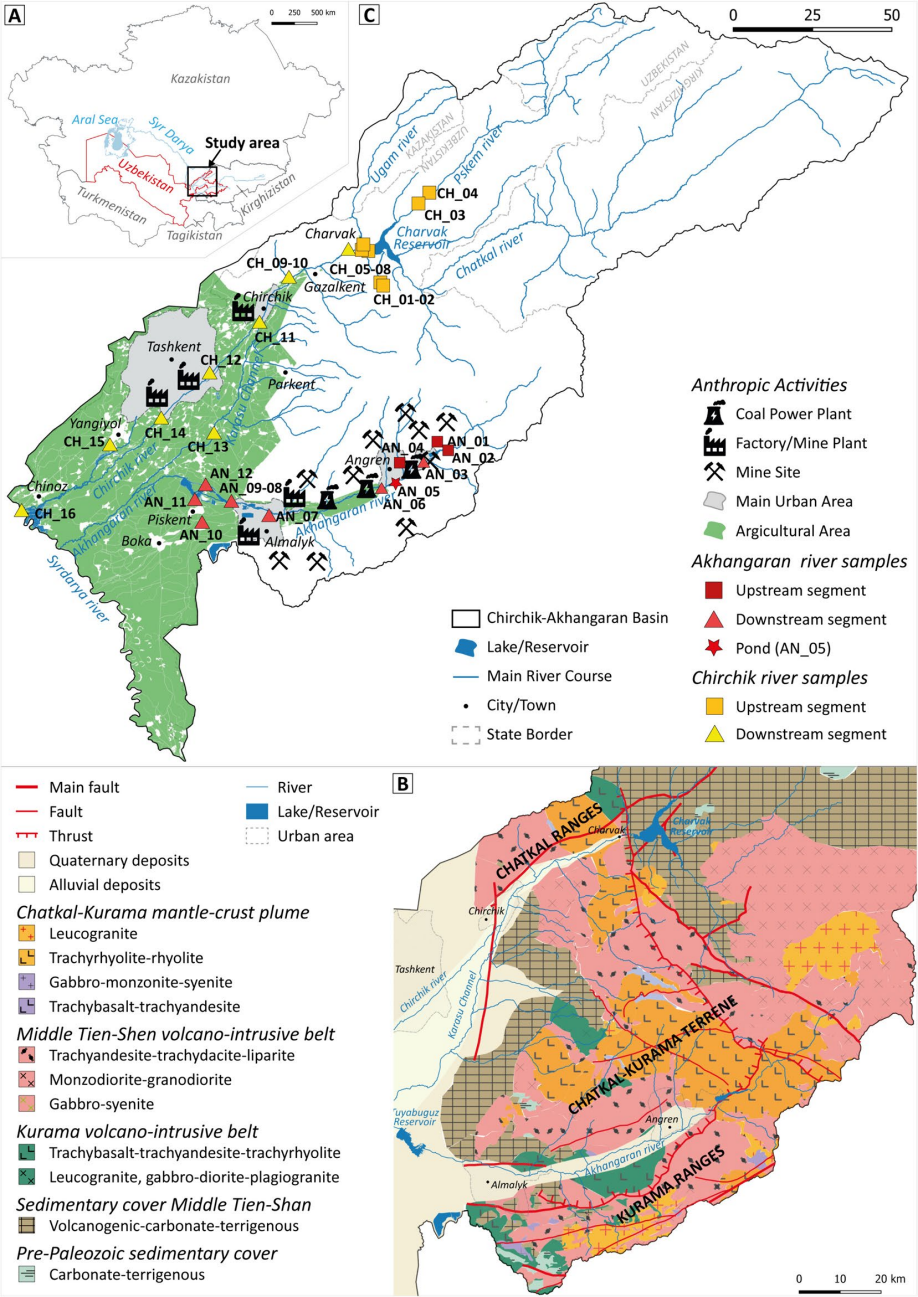
Study area. **A** Syrdarya basin location, with the study area. **B** Geological sketch map (modified after Turamuratov, 2016). **C** Sample location and land use

The CAB is located in the north-eastern part of the Republic of Uzbekistan, in the Tashkent province, between the Syrdarya river and the ridges of the Middle Tien Shan Mountains (Fig. 1A). In the north-western part, the area is bordered by the Keles river valley and the Ugam Mountain ridges. In the eastern part, the Chaktal–Kurama ridge separates the Chirchik–Akhangaran basin from the Ferghana Valley.

The Chirchik river has several tributaries, mostly from the proglacial zone and mountain-base catchments (Ugam, Koksu, Chatkal, and Pskem rivers). The river is formed by discharges of the Charvak water reservoir, used for seasonal streamflow control, irrigation, and hydropower generation. The Akhangaran river, crossing the western parts of the CAB, is the second primary water source of Tashkent. The Chirchik and Akhangaran river basins are mainly fed by snow and glacier seasonal melt (Erdanaev et al., 2015). The Karasu canal diverts part of the water of the Chirchik river downstream of the town of Chirchik, feeding a number of other canals, and connects the Chirchik and Akhangaran river valleys.

The average annual precipitation is 300 mm in the floodplain region, 300–400 mm in the mountain-base region, and 500–600 mm in the high mountain nival-glaze zone. Precipitations mostly occur in early spring and winter.

Geologically, the study area is part of the Chatkal–Kurama terrane, the western segment of the Middle Tian Shan (Fig. 1B). In the upper part of the Chirchik river, volcanogenic-carbonate-terrigenous sequences crop out together with volcano-intrusive rocks, mainly represented by leucogranite (Chatkal–Kurama mantle-crust plume), monzodiorite-granodiorite and trachyandesite-trachydacite (Middle Tien Shen units), and trachybasalt (Pre-Cambrian basement). The upper part of the Akhangaran catchment is characterized by the absence of sedimentary sequences, except for some limited outcrops in the lower part, and by a wider variety of volcano-intrusive rocks compared to the Chirchik Valley. The primary magmatic rocks vary from femic (gabbro-syenite) to acid (leucogranite). The fluvial quaternary and recent deposits cover the lower part of the CAB.

Annually, all economic sectors of the Tashkent province consume an average of 4000 mln m^3^ of water. Crop irrigation uses an average of 3200 mln m^3^ of water, and the rest is used for industry, communal, and other services (HJICA, 2011). Cotton and wheat cover about 62% of the total cultivated area (Erdanaev et al., 2022). Many industrial enterprises are present in the CAB (Fig. 1C); in some cases, insufficiently treated wastewaters are dumped into rivers (Makhmudova & Buriev, 2020). In particular, the Akhangaran valley hosts a heavy industrial complex developed during and after World War II, which includes lignite-kaolinite brown coal open-cast working, coal firing power stations, gold refineries, industries related to the processing of raw cotton, and rubber factories located near the city of Angren. These activities have led to the accumulation of significant amounts of waste exported to landfills which occupy substantial areas.

## Materials and methods

### Sampling

A total of 28 water samples, including surface water and groundwater, were collected during a survey carried out in October 2023 (low flow conditions) from the Chirchik (16 samples) and Akhangaran (12 samples) river systems (Table 1). Sampling sites are shown in Fig. 1C. Water samples were collected both in upstream catchments, with absent or low levels of anthropization, and downstream in the anthropized alluvial plain.

**Table 1:** Location of water samples

Chirchik river	Site	Lat	Lon
*Upstream segment*			
CH_01	Well in Chimgam valley	41.5449	70.0229
CH_02	Aksay stream	41.5454	70.0232
CH_03	Spring water along Pskem river	41.7448	70.1541
CH_04	Pskem river	41.7724	70.1907
CH_06	Chirchik river downstream Charvak reservoir dam	41.6279	69.9479
*Downstream segment*			
CH_05	Ugam river	41.6302	69.9475
CH_07	Spring near Charvak city	41.6266	69.9382
CH_08	Spring near Charvak city	41.6229	69.9405
CH_09	Canal upstream Chirchik city	41.5298	69.6877
CH_10	Chirchik river upstream Chirchik city	41.5296	69.6869
CH_11	Chirchik river downstream Chirchik city	41.4309	69.5828
CH_12	Chirchik river near Tashkent city	41.262	69.3762
CH_13	Karasu canal	41.1518	69.4273
CH_14	Chirchik river downstream Tashkent city	41.168	69.2308
CH_15	Well in Yangiyol city (45 m in depth)	41.0945	69.0529
CH_16	Chirchik river upstream Syrdarya confluence	40.9004	68.7092
Akhangaran river	**Site**	**Lat**	**Lon**
*Upstream segment*			
AN_01	Small tributary upstream the Angren coal mine	41.063	70.2057
AN_02	Akhangaran river upstream the coal mine	41.0539	70.2298
AN_04	Dukentsoy stream	41.0158	70.1087
*Downstream segment*			
AN_03	Infiltration pond in the vadose zone	41.0209	70.1559
AN_06	Akhangaran river downstream Angren city	40.9687	70.0522
AN_07	Akhangaran river upstream Almalyk city	40.8998	69.5989
AN_08	Akhangaran river downstream Almalyk city	40.9189	69.5154
AN_09	Akhangaran river downstream Almalyk city	40.9187	69.5149
AN_10	Piskent canal	40.8734	69.3522
AN_11	Akhangaran river upstream Tuyabuguz Reservoir	40.9353	69.3618
AN_12	Akhangaran river upstream Tuyabuguz Reservoir	40.9644	69.378
*Coal fly ash dump*			
AN_05	Pond from landfilled coal ash	40.9964	70.1043
AN_S5	Fly ash sample	40.9964	70.1043

In particular, in the Chirchik river system, five sampling stations were located in the upstream segment; two of them were springs (CH_01 and CH_03 at Chimgan and Karabulak, respectively; the first one is used for drinking purposes), while the other three represented surface waters (CH_02, CH_04, and CH_06). In addition, the spring water CH_07 (also used as drinking water) and CH_08 were located in the Charvak urban setting, downstream the Charvak Lake dam. The surface waters CH_05, CH_09, CH_10, CH_11, CH_12, CH_13, CH_14, and CH_16 were collected downstream from the base mountain to the floodplain, where groundwater was also sampled through the CH_15 water well. In the Akhangaran river system, three sampling stations AN_01, AN_02, and AN_04 (surface waters) were in the upstream segment, while most of the samples were in the floodplain, and included groundwater (AN_03) and surface water (AN_06, AN_07, AN_08, AN_09, AN_10, AN_11, and AN_12). In addition, AN_05 was collected from the pond in the landfilled coal ash basin, together with a sample of the coal fly ash (AN_S5). Waters were filtered in the field through 0.45-μm nylon filters and collected in pre-cleaned polyethylene bottles. Ultrapure nitric acid was used as a preservative for major cation and trace element analysis.

### Physico-chemical parameters and major and trace element chemistry

Temperature (*T*, °C), pH, and electrical conductivity (EC, μS/cm at 25 °C) were measured in the field using a digital thermometer and a pH/conductivity meter (Delta OHM HD 2105.1). The uncertainties were ± 0.8 °C, ± 0.02 pH unit, and ± 0.5% μS/cm, respectively. Dissolved oxygen (DO) was also measured in the field, using a DO meter (Delta OHM 2107.1 equipped with polarographic sensor); the uncertainty was ± 0.2 mg/L. Alkalinity was measured by titration using 0.1 N HCl to the endpoint of the methyl orange indicator and assigned to bicarbonate ion, assumed as the dominant species. Major cations and anions were determined by ion chromatography using a Thermo Scientific ICS-900 instrument. For anion analysis, a Dionex IonPac AS23 analytical column (4 × 250 mm) was used along with the ASRS 500 (4 mm) suppressor. For the cations, a Dionex IonPac CS12A-5 µm analytical column (3 × 150 mm) was used with the CMMS 300 (2 mm) suppressor. The relative standard deviation (RSD) (calculated on five replicate injections) was less than 5%. The coal fly ash was analyzed for trace elements after mineralization using a Milestone Ethos Easy microwave and following the USEPA Method 3052. Trace element concentrations in the Akhangaran waters and coal fly ash were determined by ICP-MS using a Perkin Elmer NexIon 300X, with ^103^Rh, ^187^Re, and ^209^Bi as internal standards to correct for signal fluctuations and matrix effects. Data accuracy and precision were tested by replicate analyses (*n* = 10) of the certified reference solutions EnviroMAT groundwater low (ES-L) and wastewater low (EU-L). At the concentrations of the reference solutions, the precision was better than 10% RSD. Deviations from the certified values were less than 5%, except for As, Be, Li, Sb, Tl, and Zn (5–10%). The Chirchik trace element analyses were performed by ICP-SFMS (ELEMENT 2, Thermo Scientific, at Luleå laboratory of ALS Scandinavia AB). Further details on measuring parameters and operation conditions are reported in Rodushkin et al. (2005). Ultrapure water (Millipore, Milli-Q, 18.2 MΩ/cm) was used for sample dilution and standard preparation.

### Mineralogical analysis

X-Ray powder diffraction analyses (XRPD) were performed using a Bruker D2 phaser diffractometer operating at 30 kV and 10 mA, equipped with a Lynxeye detector, and using Cu Kα radiation (λ = 1.54184 Å). Diffraction data were collected in the 6–60° 2θ range, with scan step 0.02° and counting time 60 s per step. The identification of mineralogical phases in the XRPD patterns was carried out through the EVABruker software (Bruker AXS, Karlsruhe, Germany) using the PDF2-2023 database.

### Geochemical modeling

Speciation and saturation index (SI) represent the extent to which water is undersaturated or supersaturated with respect to a particular mineral phase, and given by log(IAP/*K*_eq_), where IAP is the ion activity product and *K*_eq_ is the equilibrium constant for a dissolution reaction, and the stability relationships among minerals were obtained using the Geochemist’s Workbench software (release 12).

## Results

### Physico-chemical parameters

**Fig. 2:**
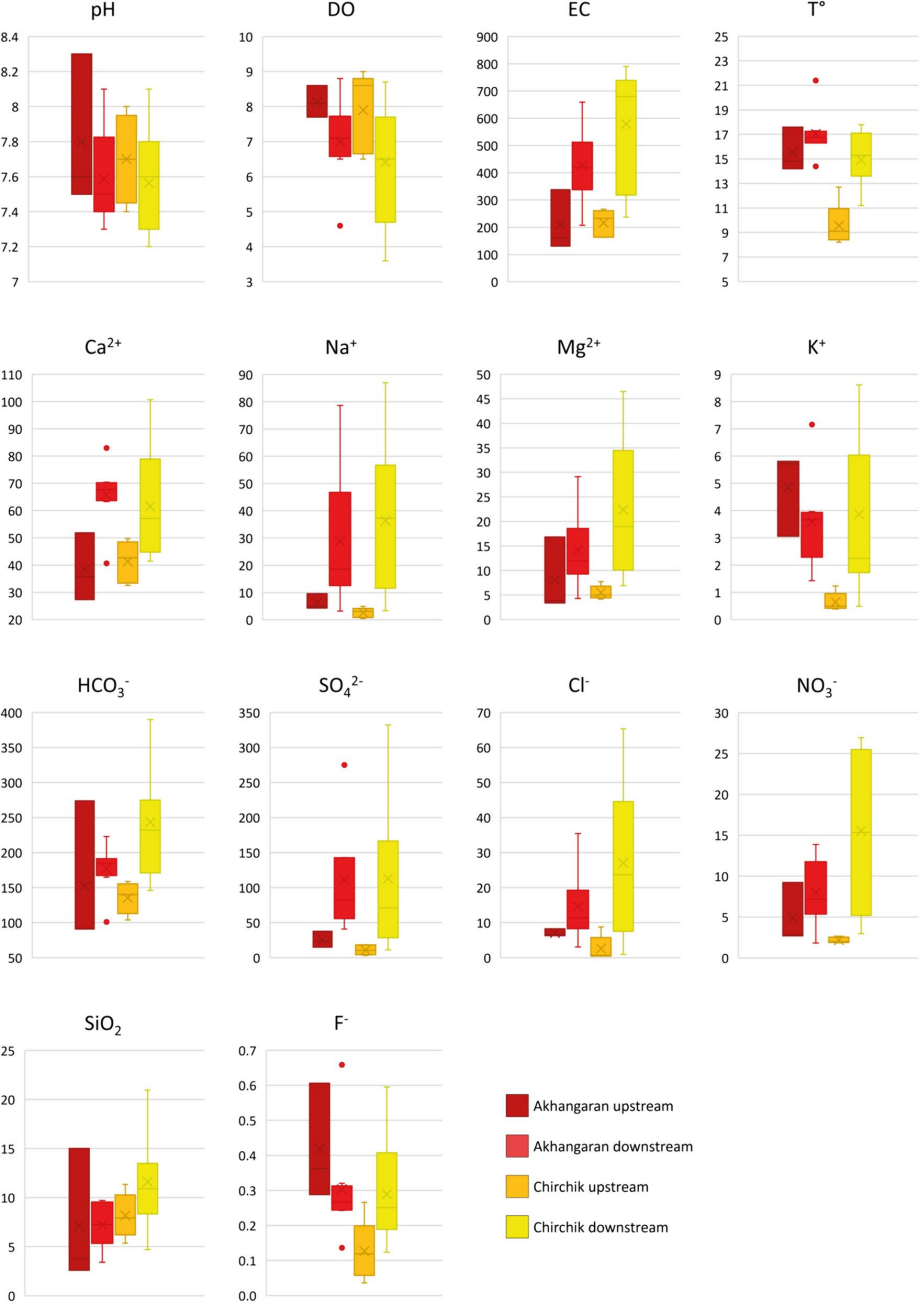
Box diagram of physico-chemical parameters and major ions. Major ion concentration and dissolved oxygen (DO) are expressed in mg/L; electrical conductivity (EC) in µS/cm, *T* in °C. AN_05 sample is not included (see text)

The physico-chemical parameters are reported in Table 2 and graphically shown through box plots in Fig. 2. The temperature was in the range 14.2–17.6 °C (av. 15.5 °C) and 8.2–13.6 °C (av. 10.3 °C) upstream, and 14.4–21.4 °C (av. 17 °C) and 13.3–17.8 °C (av. 15.4 °C) in downstream catchments from the Akhangaran and Chirchik rivers, respectively. A temperature of 22.4 °C was measured in the stagnant AN_05 water.

**Table 2:** Water-type classification, physico-chemical parameters, major ions, silica concentration, and total dissolved solid (TDS) (na = not analyzed; < LOD = limit of detection)

Sample	Water type	pH	DO	EC	*T*	Na^+^	K^+^	Mg^2+^	Ca^2+^	F^−^	Cl^−^	NO_3_^−^	PO_4_^3−^	SO_4_^2−^	HCO_3_^−^	SiO_2_	TDS
			(mg/L)	(µS/cm)	(°C)	(mg/L)	(mg/L)	(mg/L)	(mg/L)	(mg/L)	(mg/L)	(mg/L)	(mg/L)	(mg/L)	(mg/L)	(mg/L)	(mg/L)
Chirchik river																	
*Upstream*																	
CH_01	Ca-HCO_3_ type	7.7	6.8	164	9.1	3.04	0.44	5	34	0.13	0.51	2.65	< LOD	5.4	122	11.3	185
CH_02	Ca-HCO_3_ type	8	6.5	233	8.2	4.92	1.23	4.7	50	0.08	8.78	1.85	< LOD	10.5	152	7.9	242
CH_03	Ca-HCO_3_ type	7.4	8.6	255	9.2	0.45	0.38	4.2	47	0.04	0.47	2.45	< LOD	3.2	140	5.3	204
CH_04	Ca-HCO_3_ type	7.5	8.6	163	8.6	1.24	0.49	5.9	33	0.27	0.68	1.99	< LOD	18.7	104	7.1	173
CH_06	Ca-HCO_3_ type	7.9	9	266	13	3.41	0.69	7.7	43	0.12	2.78	1.93	< LOD	17.8	159	9.2	245
*Downstream*																	
CH_07	Ca-HCO_3_ type	7.2	6.2	603	15	11.6	2.15	27.8	44	0.37	11.06	26.9	< LOD	28.5	390	18.4	560
CH_08	Mg-HCO_3_ type	7.6	8.7	259	14	3.34	0.49	10.1	46	0.13	0.94	5.2	< LOD	11.4	171	9.4	258
CH_15	Ca-HCO_3_ type	7.3	3.6	733	18	37.2	3.71	46.5	41	0.59	43.13	25.7	< LOD	114	366	21	700
CH_05	Ca-HCO_3_ type	7.9	7.5	237	11	5.03	2.24	6.9	45	0.12	4.12	2.95	0.25	19.1	152	7.3	245
CH_09	Ca-HCO_3_ type	7.6	na	760	14	49	8.4	25.4	79	0.24	23.68	13.6	< LOD	167	275	13	654
CH_10	Ca-HCO_3_ type	7.8	na	704	13	31.91	1.99	18.9	66	0.26	13.96	10.7	< LOD	102	232	12.2	490
CH_11	Ca-HCO_3_ type	8.1	na	318	14	12.26	1.68	9.7	46	0.19	7.56	4.6	< LOD	51.8	146	8.3	288
CH_12	Na-SO_4_ type	7.2	na	550	16	56.71	8.61	12.7	71	0.41	65.34	25.5	1.03	67.2	207	4.7	520
CH_14	Ca-HCO_3_ type	7.5	6.5	680	17	38.11	6.03	15.2	57	0.21	44.57	25	< LOD	71.1	262	13.5	533
CH_16	Mg-HCO_3_ type	7.6	4.7	790	17	67.86	5.49	38.4	101	0.41	46.21	15.3	< LOD	274.6	269	8.8	827
CH_13	Ca-SO_4_ type	7.4	7.7	739	15	87	1.72	34.5	81	0.25	37.38	15.4	< LOD	332.1	207	10.9	808
Akhangaran river																	
*Upstream*																	
AN_01	Ca-HCO_3_ type	8.3	7.7	338	14	9.54	5.71	16.8	52	0.61	6.64	9.21	< LOD	37.7	274	15	427
AN_04	Ca-HCO_3_ type	7.5	8.1	162	18	4.89	5.8	3.9	36	0.29	8.22	2.83	< LOD	23.9	92	3.8	181
AN_02	Ca-HCO_3_ type	7.6	8.6	131	15	4.28	3.06	3.4	27	0.36	6.29	2.69	< LOD	15	91	2.6	156
*Downstream*																	
AN_03	Ca-HCO_3_ type	7.4	4.6	207	21	3.19	7.15	4.3	41	0.66	3.09	1.83	0.69	40.9	101	3.4	207
AN_06	Ca-HCO_3_ type	7.6	8.8	331	17	11.45	3.82	11.4	70	0.24	9.65	12.1	< LOD	51	189	7.5	366
AN_07	Ca-HCO_3_ type	8.1	7.9	357	17	20.84	3.96	12.5	66	0.32	13.12	8.39	< LOD	87.6	180	9.1	402
AN_08	Ca-HCO_3_ type	7.3	6.5	433	17	16.25	2.07	9.9	70	0.29	9.07	5.26	< LOD	78	189	9.7	389
AN_09	Ca-HCO_3_ type	7.4	6.8	407	17	15.79	1.43	9.1	63	0.26	8.07	5.69	< LOD	70	165	6.3	345
AN_10	Ca-SO_4_ type	7.6	7.1	659	14	78.67	2.95	29.1	83	0.14	35.42	13.9	< LOD	275	174	5	697
AN_11	Ca-HCO_3_ type	7.9	7.2	514	16	51.05	3.5	18.7	70	0.25	18.77	5.97	< LOD	143	192	7	511
AN_12	Ca-HCO_3_ type	7.4	7.1	505	16	33.81	3.85	18.3	65	0.27	19.39	10.9	< LOD	142	223	9.7	525
*Coal fly-ash water*																	
AN_05	Ca-SO_4_ type	10.1	7.1	819	22	8.66	8.62	3.4	221	6.66	7.25	4.28	< LOD	522	92	4.4	878

In the Akhangaran river system, pH ranged from 7.5 to 8.3 and from 7.3 to 8.1 in upstream and downstream catchments, respectively. The Chirchik pH ranged from 7.4 to 8.0 and from 7.2 to 8.1 upstream and downstream, respectively. No significant differences in pH were observed between surface and groundwater. The measured pH ranges are consistent with dissolved carbonate equilibria and atmospheric or soil carbon dioxide in open systems, and/or the contribution of groundwater with higher pCO_2_. The water sample AN_05, i.e., the sample from the coal ash disposal site, was characterized by a pH value of 10.1.

The dissolved oxygen (DO) concentration ranged between 7.7 and 8.6 mg/L and between 4.6 and 8.8 mg/L in the Akhangaran river system, and between 6.5 and 9 mg/L and between 3.6 and 7.7 mg/L upstream and downstream, respectively, in Chirchik. The DO in water from the upstream catchment was somewhat lower compared with what expected for air-saturated water at the proper temperature; in addition, the average dissolved oxygen concentration decreased significantly in lowland waters compared with the upstream catchments, possibly reflecting the effects of nutrient inputs.

In waters from both the Akhangaran and Chirchik basins, the electrical conductivity (EC) showed a considerable increase in floodplain compared with the upstream catchments, increasing from 164–338 to 207–659 µS/cm at Akhangaran and from 163–266 to 318–790 µS/cm at Chirchik. The AN_05 sample deviated toward the highest EC (819 µS/cm).

### Major ions and trace elements

**Fig. 3:**
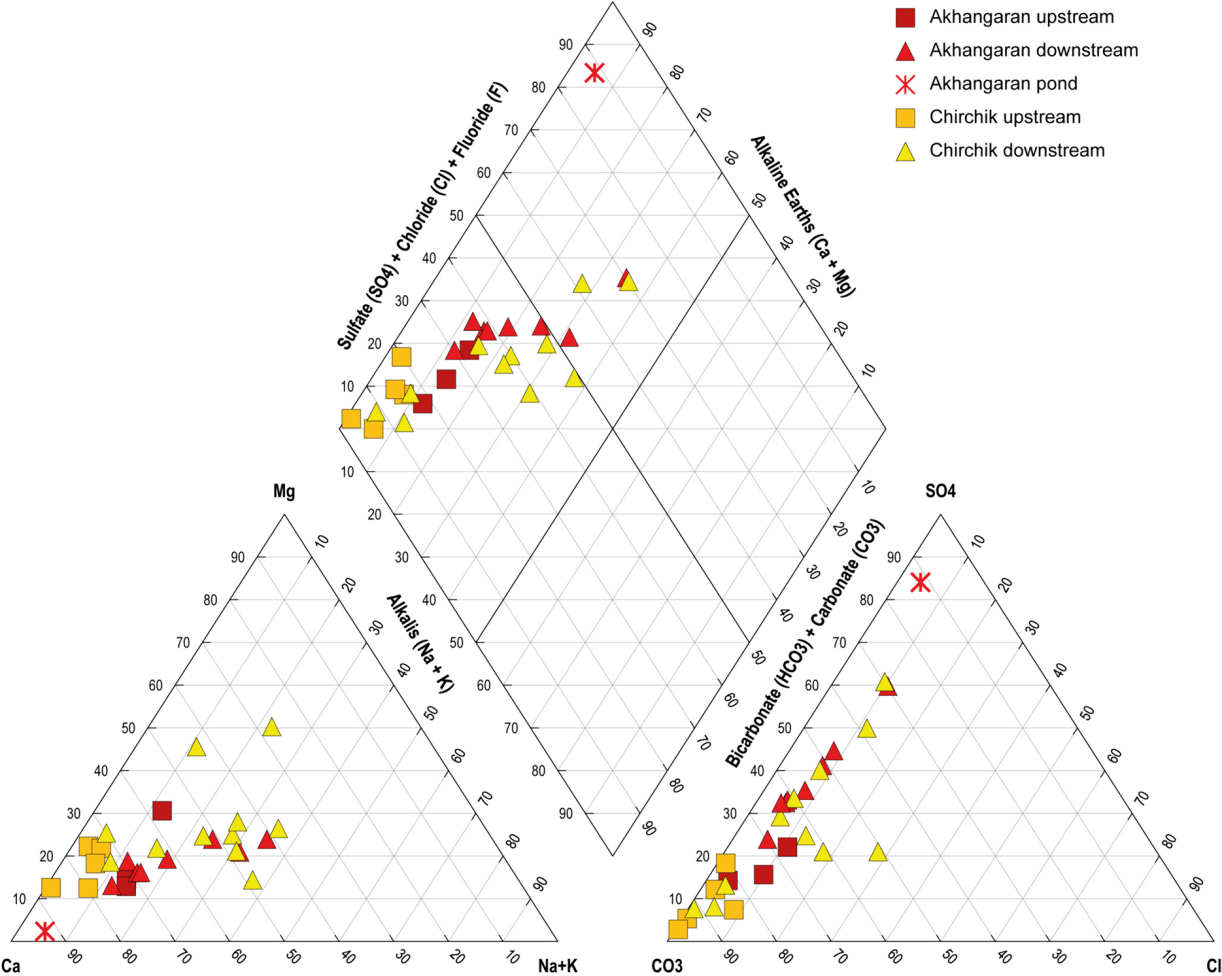
Piper diagram for Chirchik and Akhangaran water samples. Symbols are in the legend

The major ion concentrations and calculated water type are presented in Table 2, and portrayed graphically by box plots in Fig. 2. The proportions (in equivalents) of the major cations and anions are reported in the Piper diagram of Fig. 3 (Piper, 1944). Most waters from Akhangaran and Chirchik catchments belong to the Ca-HCO_3_ hydrofacies. This suggests that Ca^2+^ and HCO_3_^−^ are the major constituent cations and anions despite the dominance of silicate rock outcrops. Exceptions are AN_5, AN_10: Ca-SO_4_ type; CH_7, CH_15: Mg-HCO_3_ type; CH_13: Na-SO_4_ type; and CH_16: Ca-SO_4_ type.

Waters collected in the lowlands are distinguishable from the headwaters, clearly showing a spatial pattern for major ions (Fig. 3). In particular, waters from both the Akhangaran and Chirchik river systems showed an increase in Na^+^, K^+^, SO_4_^2−^, and Cl^−^ downstream, compared with the upstream catchment. In addition, Mg^2+^, NO_3_^−^, and HCO_3_^−^ in Chirchik markedly increased downstream. These changes likely reflect the contribution of soil water-soluble salts.

**Fig. 4:**
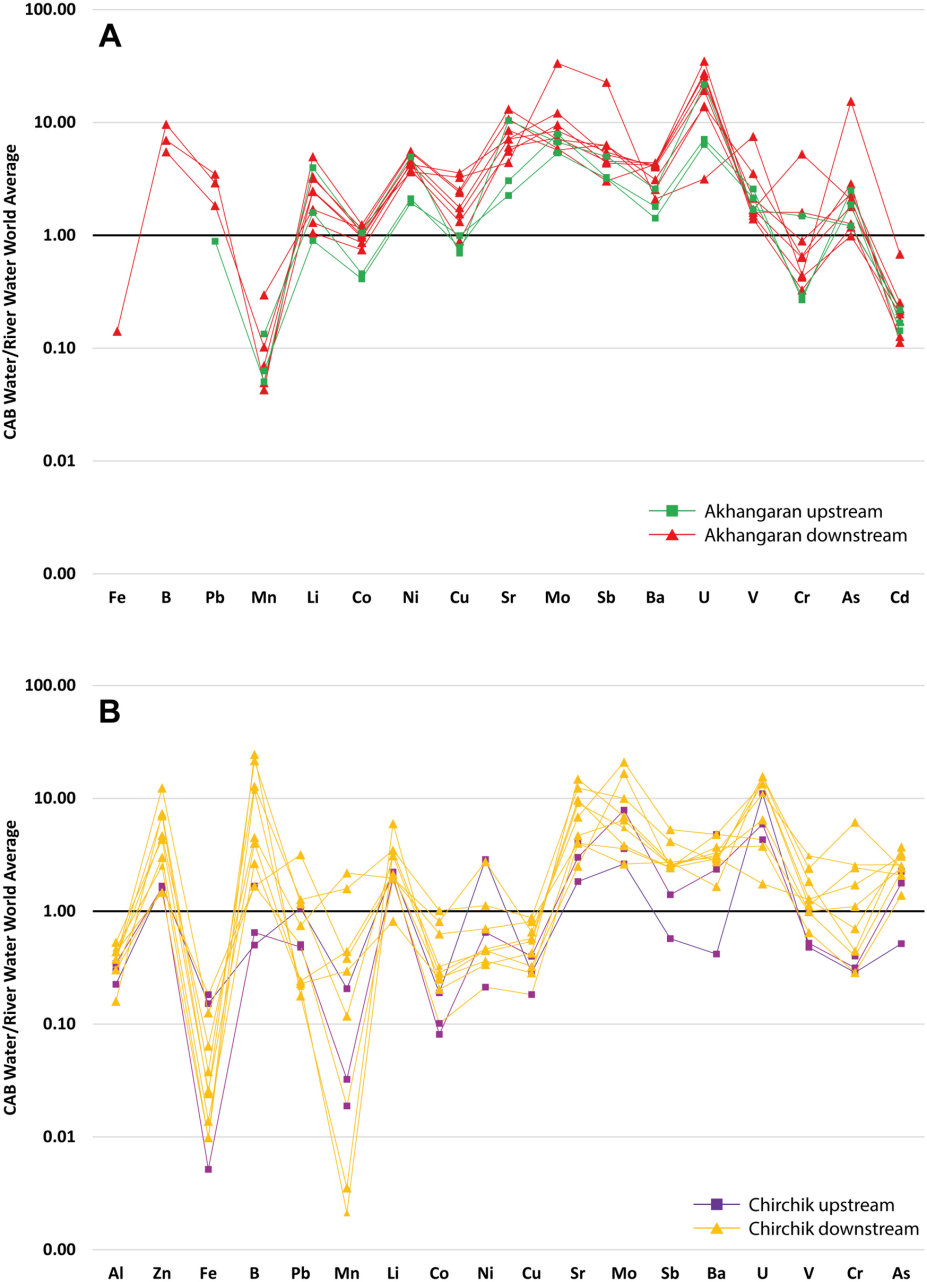
Trace element data in surface water samples normalized to the average concentration value in river water worldwide (Gaillardet et al., 2014): (**A)** Akhangaran (Al and Zn below detection limits); (**B**) Chirchik (Cd below detection limit)

Trace element analyses are reported in Table 3. The concentration of potentially toxic elements (PTEs) in the CAB waters was lower than the thresholds established for water quality by different regulations (WHO, 2022; EU, 2020; USEPA, 2019) and, in some cases, even below the detection limit. An exception is represented by sample AN_05, which was characterized by Al, B, Mo, Sb, and As concentration exceeding the regulatory limits of 200, 1000, 40, 5, and 10 µg/L, respectively, established by EU and USEPA (USEPA, 2019; WHO, 2022). However, despite being present in low concentration in most waters, some trace elements showed higher concentrations than the averaged values reported for rivers worldwide (Gaillardet et al., 2014); in particular, waters from both river systems were relatively enriched in B, Li, Sr, Mo, Sb, Ba, V, As, and U. In addition, Akhangaran and Chirchik showed higher Ni, Cu and Zn, Cr content (Fig. 4).

**Table 3:** Trace element concentration (µg/L) (na = not analyzed)

Sample	Li	Be	B	Al	Mn	Co	Ni	Cu	Zn	Sr	Mo	Ag	Sn	Cd	Sb	Ba	Tl	Pb	Th	U	V	Cr	Fe	As
Chirchik river																								
*Upstream*																								
CH_01	6.1	< 0.02	7.2	8.6	0.24	0.008	< 0.1	0.14	0.49	170	3.3	< 0.01	< 0.1	< 0.05	0.03	9.4	0.005	0.014	< 0.005	6.2	1.1	0.8	0.45	0.63
CH_02	4.1	< 0.02	5.1	7.2	7	0.03	2.3	0.42	1	110	1.1	< 0.01	< 0.1	< 0.05	0.04	9.6	0.007	0.084	< 0.005	4.1	0.34	0.2	10	0.32
CH_03	0.9	< 0.02	< 5	12	0.24	0.01	< 0.1	0.51	2.7	69	0.06	< 0.01	< 0.1	< 0.05	0.02	9.8	0.009	0.038	< 0.005	0.31	0.44	0.21	0.38	0.76
CH_04	3.7	< 0.02	6.6	11	1.1	0.01	0.52	0.59	0.97	180	3.3	< 0.01	< 0.1	< 0.05	0.1	54	0.009	0.038	< 0.005	2.2	0.37	0.22	0.34	1.1
CH_06	3.5	< 0.02	17	9.5	0.64	0.02	0.17	0.27	0.97	240	1.5	< 0.01	< 0.1	< 0.05	0.17	110	0.011	0.04	< 0.005	1.6	0.69	0.28	12	1.4
*Downstream*																								
CH_05	3.6	< 0.02	17	12	13	0.09	0.56	1.2	7.4	240	1.1	< 0.01	< 0.1	< 0.05	0.19	69	0.014	0.25	< 0.005	0.65	0.9	0.49	8.3	2.0
CH_07	9.6	< 0.02	49	7.8	0.11	0.03	0.14	0.4	1.5	840	2.8	< 0.01	< 0.1	< 0.05	0.11	130	0.013	0.021	< 0.005	3.8	3.4	3.5	< 0.25	2.4
CH_08	4.5	< 0.02	25	7.7	0.41	< 0.008	< 0.1	0.16	1.8	290	0.67	< 0.01	< 0.1	< 0.05	0.05	62	0.011	0.054	< 0.005	1.4	0.65	0.78	3.1	1.7
CH_09	5.7	< 0.02	220	12	4	0.04	0.37	0.85	4.2	580	1.6	0.016	< 0.1	< 0.05	0.17	67	0.042	0.092	< 0.005	5	0.8	1.7	1.6	1.3
CH_10	6.2	< 0.02	120	15	0.07	0.05	0.35	0.8	1.5	540	2.3	< 0.01	< 0.1	< 0.05	0.18	76	0.01	0.017	< 0.005	4.1	2.2	1.8	0.64	1.6
CH_11	3.9	< 0.02	41	17	15	0.04	0.29	0.42	2.6	280	2.7	< 0.01	< 0.1	< 0.05	0.18	72	0.015	0.019	< 0.005	2.4	0.71	0.77	1.7	1.5
CH_12	1.5	< 0.02	27	9.7	10	0.04	0.36	0.48	1.8	150	7	0.033	< 0.1	0.05	0.17	85	0.024	0.018	< 0.005	1.4	0.46	0.2	0.91	0.86
CH_13	11	< 0.02	250	14	0.12	0.03	0.27	0.63	0.88	890	2.9	< 0.01	< 0.1	< 0.05	0.19	38	0.01	0.014	< 0.005	5.8	1.7	4.3	0.65	1.5
CH_14	3.6	< 0.02	46	12	74	0.12	2.2	0.97	2.8	410	8.8	0.013	< 0.1	0.09	0.37	110	0.023	0.059	< 0.005	5	0.91	1.2	4.2	1.9
CH_15	23	< 0.02	120	9	0.76	0.08	0.37	2.2	9.3	1600	7.3	0.071	1.2	0.09	0.11	57	0.021	0.068	< 0.005	13	2.5	5.9	0.98	0.87
CH_16	6.4	< 0.02	130	5.1	54	0.15	0.9	1.3	4.4	740	4.2	0.01	< 0.1	0.05	0.29	63	0.017	0.1	< 0.005	5.8	1.3	0.31	2.5	2.3
Akhangaran river																								
*Upstream*																								
AN_01	7.3	< 0.02	< 42	< 46	1.72	0.16	4	1.03	< 16	623	2.8	< 0.5	< 0.07	0.02	0.35	59	0.02	0.07	< 0.008	8	1.2	1.04	< 2.5	0.75
AN_02	1.65	< 0.02	< 42	< 46	2.1	0.06	1.55	1.47	< 16	135	2.3	< 0.7	< 0.07	0.01	0.22	33	0.02	< 0.1	0.006	2.4	1.52	0.2	< 2.5	1.16
AN_04	2.9	< 0.02	< 42	< 46	4.5	0.07	1.69	1.16	< 16	183	3.3	< 0.5	< 0.07	0.01	0.23	41	0.03	< 0.1	< 0.008	2.6	1.83	0.19	< 2.5	1.55
*Downstream*																								
AN_03	5.9	< 0.02	98	< 46	< 0.9	0.17	2.9	4.8	< 16	266	14	< 0.5	< 0.07	0.05	1.59	48	0.01	0.23	0.08	1.2	5.3	0.31	9.3	9.6
AN_06	4.5	< 0.02	56	< 46	3.5	0.14	3.4	2.3	< 16	429	5.1	< 0.5	< 0.07	0.02	0.36	96	0.01	0.15	< 0.008	8.4	1.51	0.62	< 2.5	1.44
AN_07	4.5	< 0.02	71	< 46	1.68	0.15	3.4	5.3	< 16	427	3.5	< 0.5	< 0.07	0.02	0.38	93	0.01	0.28	< 0.008	7.1	1.21	0.45	< 2.5	1.11
AN_08	2.4	< 0.02	< 84	< 92	< 0.9	0.13	3.5	1.32	< 16	363	3.1	< 0.5	< 0.07	0.02	0.32	101	< 0.01	< 0.2	< 0.008	9.7	0.99	0.23	< 5	0.73
AN_09	1.94	< 0.02	< 84	< 92	< 0.9	0.11	3	1.96	< 16	334	4	< 0.5	< 0.07	0.02	0.31	96	< 0.01	< 0.2	< 0.008	10.2	1.07	0.3	< 5	0.61
AN_10	9.1	< 0.02	< 84	< 92	1.45	0.18	4.5	3.7	< 16	787	3	< 0.5	< 0.14	0.01	0.44	72	< 0.02	< 0.3	< 0.02	5.1	1.19	3.7	< 7	1.29
AN_11	6.1	< 0.02	< 84	< 92	2.4	0.16	3.7	2.6	< 16	511	2.4	< 0.5	< 0.07	0.01	0.44	59	< 0.01	< 0.2	< 0.008	5.2	2.5	0.46	< 5	1.77
AN_12	3.1	< 0.02	< 84	< 92	10.1	0.17	4.3	3.5	< 16	644	2.5	< 0.5	< 0.07	0.01	0.21	98	0.01	< 0.2	< 0.008	13	1.14	1.12	< 5	0.79
*Coal fly-ash water*																								
AN_05	116	< 0.02	2736	4787	2.2	0.43	11.1	15.4	< 23	3580	55.6	< 0.7	< 0.14	0.14	17.8	121	0.05	1.03	< 0.02	1.5	227	2.8	< 10	80
*Coal fly ash (mg/kg)*																								
AN_S5	99	7.9	na	na	645	13.7	14.4	48	585	742	4.4	0.81	5.2	2.5	4.6	3315	2.6	132	34	13.3	143	38	na	104

### Saturation indices

Speciation calculations indicated that waters from the Chirchik upstream catchments were super-saturated with Mn–Fe oxyhydroxides, smectite group clay minerals (nontronite-beidellite), mica-type clay minerals illite-muscovite, zeolite group clinoptinolite, pyrophyllite, K-feldspar, and epidote; from super-saturated to under-saturated with dolomite; super-saturated to slightly under-saturated with quartz; saturated to slightly under-saturated with calcite; strongly under-saturated with gypsum and halite. In the Akhangaran upstream catchment, waters resulted to be strongly super-saturated with Mn–Fe oxyhydroxides; supersaturated with quartz; super-saturated to slightly under-saturated with dolomite and calcite. Mineral saturation data indicated that the chemical composition of water in upstream CAB catchments was primarily controlled by local geology and water–rock interactions. In particular, the water chemical evolution is mostly driven by silicate weathering, including Fe-bearing minerals that may form oxyhydroxides as weathering products, and carbonate dissolution. Evaporite (i.e., halite) dissolution is not a significant hydrogeochemical process. In the downstream Chirchik river system, the water became less undersaturated in gypsum and halite, which was produced by an increase of sulfate and chloride, not related to geogenic sources. The mineral saturation state in the water sample AN_05 collected in the coal fly-ash pond differed markedly. This sample was super-saturated with antigorite, amesite, talc, Mn–Fe oxides (birnessite, todorokite, hematite), tremolite, andradite, smectite group clay minerals, clinochlore, epidote, pyrolusite, hausmannite, calcite, and dolomite; saturated with K-feldspar and kaolinite; and under-saturated with gypsum.

## Discussion

### Water geogenic solute

At the catchment scale, the reactivity to chemical weathering and the efficiency in solute production strongly depend on the nature of the exposed lithologies (e.g., Meybeck, 1987). Silicates and carbonates weather differently and have a different role in controlling water chemistry (Fan et al., 2014). The incongruent dissolution of the primary Al–silicate minerals that characterize igneous rocks causes the release of cations to water and the precipitation of secondary mineral phases, such as clays, oxyhydroxides, and carbonates (White et al., 1999). Igneous basic rock series and acidic volcanic rocks (e.g., rhyolites) are generally more susceptible to weathering compared with intrusive rocks, such as granites, and metamorphic rocks like schists. The rate of mineral weathering, the nature of authigenic phases that form, and the solute flux to surface waters also depend on the rate of erosion and hydrological conditions, and reflect the dilution dependence from runoff and how the solute is taken up by the biomass and adsorbed on organics and/or solid surfaces (e.g., Gabet et al., 2006; Gibbs & Kump, 1994; Maher, 2011; White & Blum, 1995; Wild et al., 2022). The sequence of the degradation process of silicates is traced by the increase in dissolved silica.

**Fig. 5:**
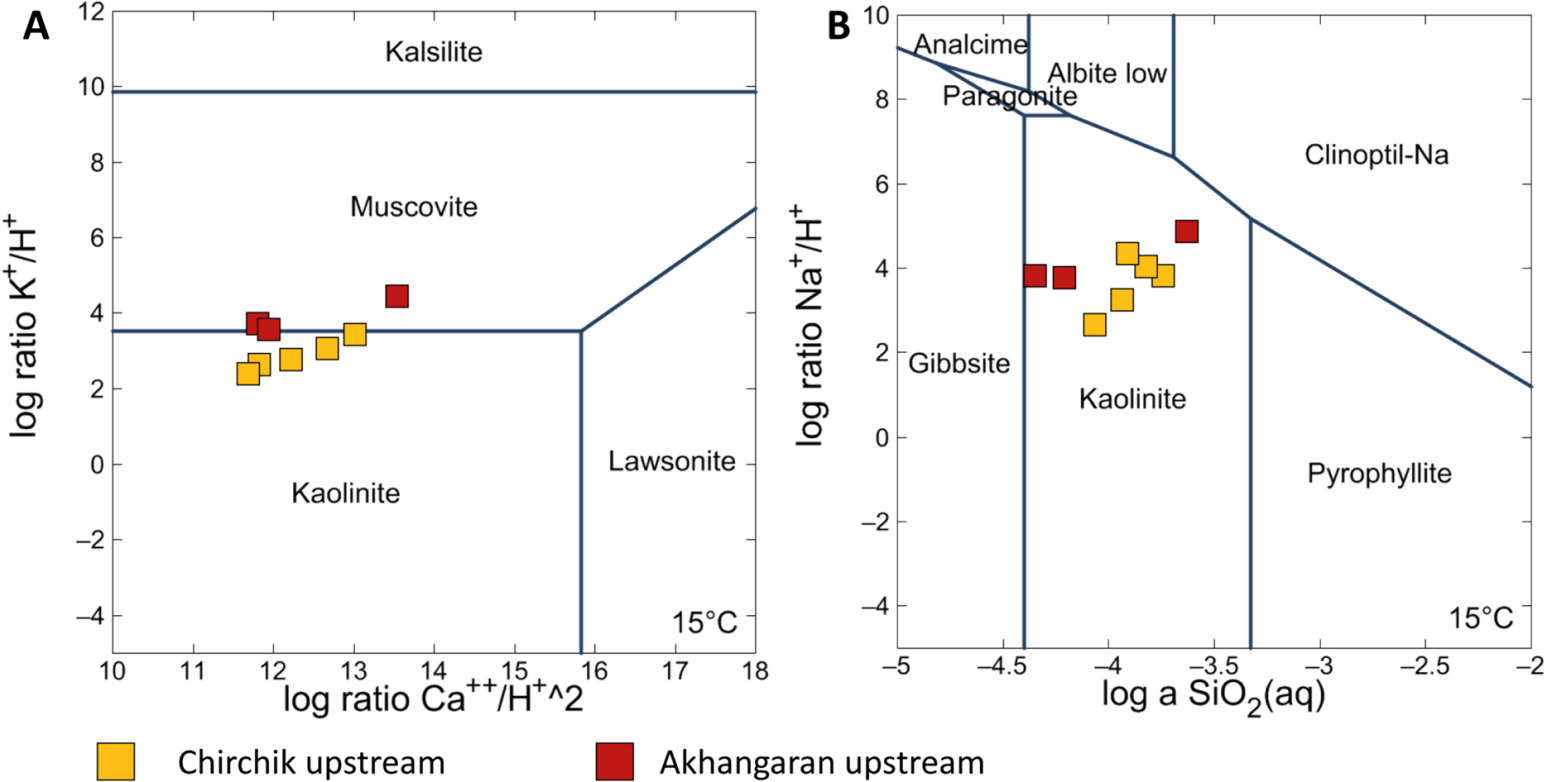
Equilibrium phase diagram. **A** Log ratio K^+^/H^+^ vs. log ratio Ca^2+^/H^+^; **B** log ratio Na^+^/H^+^ vs. log a SiO_2_(aq). Only upstream samples are considered

Waters from the upstream catchments of CAB were characterized by low nitrate content (1.85 < NO_3_^−−^ < 2.83 mg/L except for AN_01 crossing a family-based poultry: NO_3_^−−^ = 9.21 mg/L). It can therefore be expected that the dissolved load composition mostly reflects geogenic sources. The silicate stability diagrams for feldspars and related weathering products are shown in Fig. 5A and B. Despite limitations in representing real systems, stability relationships highlight that the studied waters plot prevalently in the kaolinite field, showing a possible reaction pathway to form more stable secondary phases such as muscovite.

**Fig. 6:**
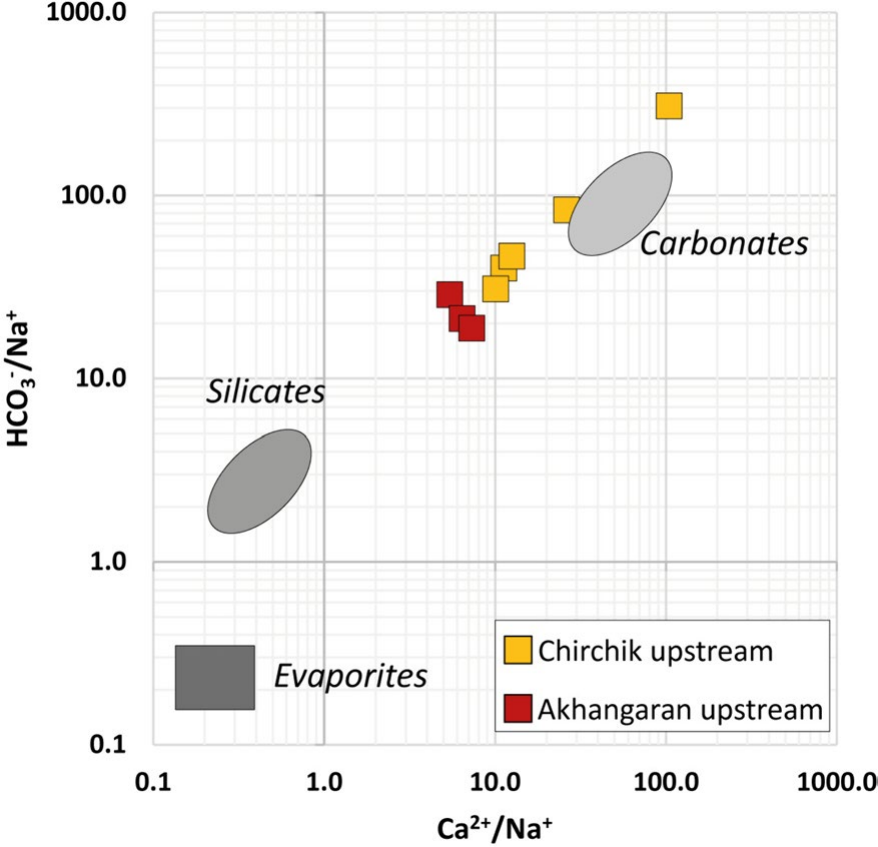
Mixing diagram HCO_3_^−^/Na^+^ vs. Ca^2+^/Na^+^. Only upstream samples are considered

In spite of the fact that volcano-plutonic rocks are the major exposed lithologies in the basins, waters from upstream catchments belong to the Ca-HCO_3_ type, suggesting that carbonate weathering has an important role in controlling the riverine Ca and HCO_3_ fluxes. Indeed, pedogenetic calcite precipitation is favored by the release of Ca^2+^ and HCO_3_^−^ ions during Ca-silicate weathering, and the dissolution of disseminated CaCO_3_, besides a possible role of carbonate sequences, might contribute to the observed hydrochemical response of the catchments. The hypothesis of a contribution from carbonates to water chemistry is highlighted by the Na-normalized mixing diagrams of Fig. 6, where disseminated calcite is included in the carbonate end-member (Gaillardet et al., 1999).

**Fig. 7:**
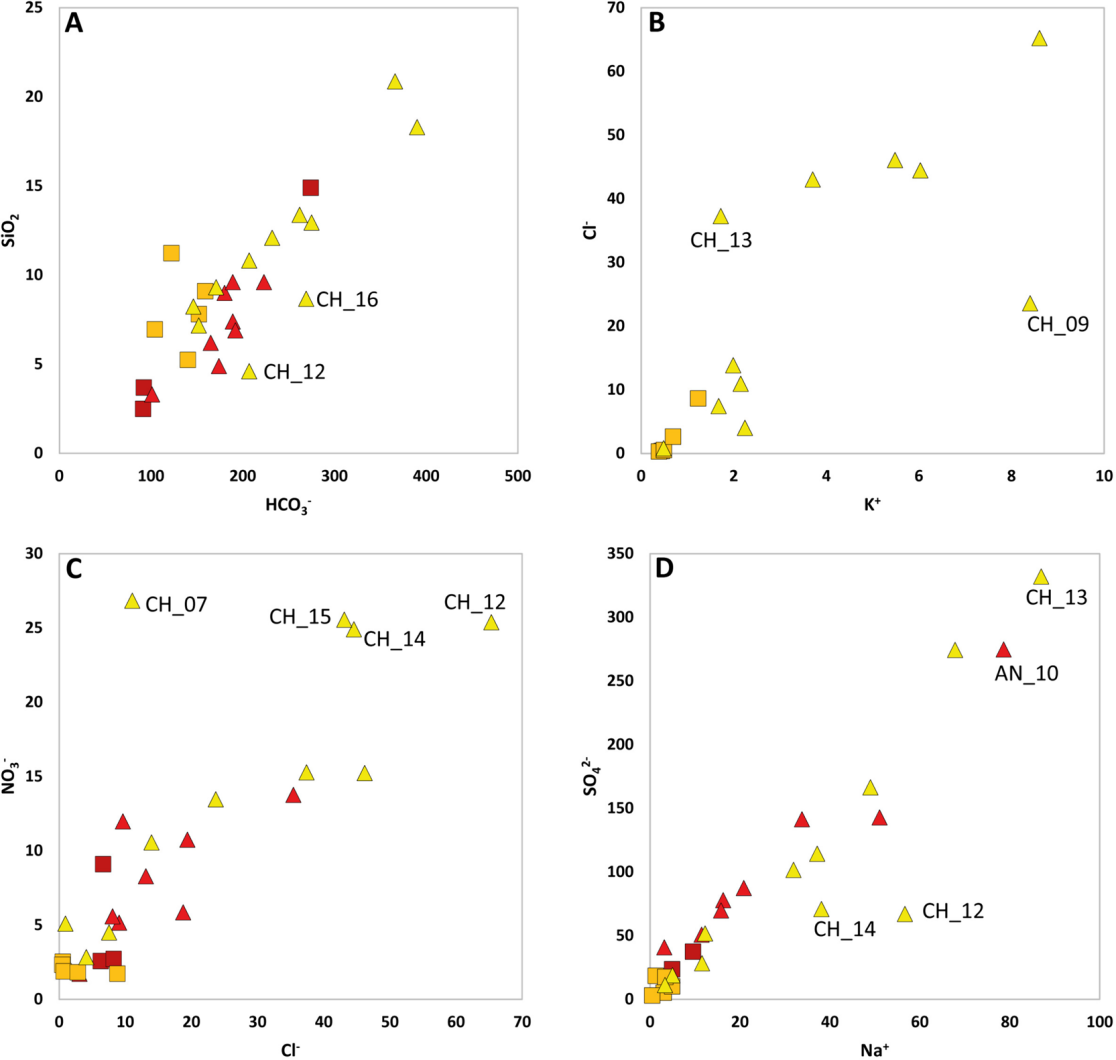
Scatter plots of HCO_3_^−^ vs. SiO_2_ (**A**); K^+^ vs. Cl^−^ (**B**); Cl^−^ vs. NO_3_^−^ (**C**); Na^+^ vs. SO_4_^2−^ (**D**) highlighting the possible contribution of anthropogenic sources to water chemistry (see text). In (**B**), only Chirchik samples are shown. Legend in Fig. 3

Despite some scatter, silica positively correlates with alkalinity (Fig. 7A). The observed cumulative trend corroborates the hypothesis of weathering profiles from silicate-dominated systems. Two samples from the Chirchik river (CH_12 and CH_16) deviated from the main trend. This might reflect the influence of algal activity such as diatom uptake in temporary stagnant water zones in segments of the impounded river (Wang et al., 2013). This hypothesis deserves further investigation. The geogenic control on water chemistry represents a baseline for water quality assessment in the lower course of the Chirchik and Akhangaran rivers.

### Impacts from the cropping system

The anthropogenic influence on water chemistry can be traced through the chloride content, since Cl^−^ is usually very low in carbonate and silicate rocks, and through the nitrate amount. Surface and groundwater from the Chirchik river system showed an increase in K^+^ downstream, compared with geogenic sources, coupled with an increase in Cl^−^ (Fig. 7B). Sample CH_09, collecting water from a diversion of the Chirchik river, deviated from this trend.

The observed pattern likely reflects the impact of the historical application of potash fertilizer KCl (Buvaneshwari et al., 2020; Tyner et al., 2000), which represents a source of water salinization in the Chirchik agricultural catchment. This correlation was not observed for waters from the Akhangaran river system. Chloride also correlated with NO_3_^−^ (Fig. 7C), particularly in the downstream segment of Chirchik, for both surface and groundwater. This observation suggests that nitrate infiltrated at depth. The nitrogen distribution in the Chirchik river has been investigated by several authors (e.g., Nasedjanov et al., 2012; Makhmudova & Buriev, 2020; Akhmedova et al., 2020; Karimov et al., 2020). Both NO_3_^−^ and Cl^−^ in water are commonly related to contamination of the land surface by manure spreading and agricultural activities (Kohn et al., 2016); the observed correlation suggests that nitrate behaves conservatively and that conditions are not appropriate for a significant attenuation of pollution by denitrification. It should also be noted that the Chirchik river near Tashkent (CH_12, CH_14, and CH_15 samples) was characterized by the highest NO_3_^−^ concentration, indicating the potential contribution of urban runoff (Zendehbad et al., 2019; Zhang et al., 2022), including fertilizer release by road dust during street sweeping. An additional source for nitrate contamination in this segment of the Chirchik river might be represented by the Chirchik–Maxsam enterprise that produces nitrogenous fertilizers (Karimov et al., 2020).

A positive correlation was observed for most waters, from both the Chirchik and Akhangaran basins, between the SO_4_^2−^ and the Na^+^ content (Fig. 7D), with the increase in sulfate and sodium content in the floodplain waters compared with the upper catchments. Readily soluble sodium bisulfate (NaHSO_4_) has been widely applied by the broiler industry in poultry litter treatments to reduce NH_3_^+^ volatilization, lowering litter pH, and inhibit microbial activity (Chadwick et al., 2020; Joerger et al., 2020), and the treated litter is applied as crop nutrients. Indeed, agricultural activities and livestock farming, including poultry farming, are highly developed in the Chirchik district (Karakulov et al., 2023). The data indicate that, in particular in the Chirchik river lowlands, intensive agricultural activities for crops and livestock production released significant amounts of fertilizers into soils formed from the different parent rocks. Irrigation and runoff led to the leaching of nutrients and other ions from the soil, resulting in an increased concentration in surface and groundwater bodies.

### Impacts from industrial activities

The industrial enterprises in the CAB (Fig. 1C) have a long-lasting heritage. In the Chirchik river catchment, it has been reported that the oil and chemical industry wastewater discharges and uncontrolled waste dumping have caused high levels of pollution. Nevertheless, as already stressed, the EPT concentrations in waters from the Chirchik river system measured in the present study are below guideline levels (USEPA, 2019; EU, 2020; WHO, 2022). It is, however, worthy of note that CH_14 and CH_16 samples showed a relatively high Mn content compared with the remaining samples, even if within the threshold for irrigation water quality (Bortolini et al., 2018). In addition, an increase in the molybdenum content was observed in samples CH_12 and CH_14, where the Chirchik river flows near Tashkent, indicating wastewater containing Mo as the possible anthropogenic source.

The Almalyk Mining and Metallurgical Complex have negative impacts on the Akhangaran river system. Previous studies (Kodirov & Shukurov, 2009; Pen-Mouratov et al., 2014; Shukurov et al., 2005, 2009) demonstrated the occurrence of metal pollution (Zn, Cd, and Pb) in the upper soil layer along the Akhangaran river downstream from the Angren industrial complex. Still, the concentration of PTEs measured in surface water was below the detection limit or lower than the threshold imposed by regulations, suggesting that contaminants eventually leached from the polluted soil are mixed and diluted in the river water. The relatively high U and Mo concentration measured in the AN_04 water sample, collected where the Dukentsoy stream enters the Chirchik river, likely reflect the occurrence of U–Mo deposits and their past exploitation upstream of the Dukentsoy valley (Tsukatani et al., 2008). In the Akhangaran valley, in the suburbs of the city of Angren, there are the Angren and Novo-Angren coal-fired power stations, and, about 5 km to the North-East, there is the Angren lignite-kaolin open-air mine. The coal fly ashes from the thermal power are stored in a large fly settling containment pond, where water evaporates through solar radiation and winds and possibly to infiltrates. X-Ray powder diffraction analysis on coal ashes indicates that quartz is the major mineralogical components of coal ashes, with calcite, muscovite, mullite (Al_5.65_ Si_0.35_ O_9.175_), hematite, and possibly rutile and anatase (TiO_2_) present in minor amounts, as reported for this kind of products (Saikia et al., 2021). The pH of the AN_05 water, collected in the ash pond, was highly alkaline: this likely reflects the hydration reaction of lime formed by the thermal decomposition of calcite and the onset of calcite precipitation, as indicated by supersaturation with CaCO_3_. This water belongs to the Ca-SO_4_ hydrofacies, the relatively high sulfate content being attributable to the dissolution of desulfurization products. In addition, the calcium/sulfate molar ratio close to unity suggests that calcium possibly originated from gypsum/anhydrite dissolution, indicating the exhaustion of the lime pool with time. Coal fly ashes are generally classified as non-hazardous waste materials in the EU. In the USA, the USEPA released the rules for coal combustion residual safety disposal (USEPA, 2015), and is increasing the concern that coal fly ashes may be enriched in PTEs with respect to the parental coal (Akar et al., 2012; Catalano et al., 2012; Chen et al., 2024). The geochemistry of the collected coal fly-ash sample indicated a high concentration of PTEs (Table 3), among which As, V, Zn, Mn, and Pb were above 100 mg/kg. Therefore, this material represents a potential source of contamination for pond water. Indeed, the AN_05 water was characterized by concentrations of As, Al, and B exceeding the threshold reported by Uzbekistan regulation (50, 200, and 500 µg/L, respectively); furthermore, the Sb concentration exceeded the 5 and 3 µg/L acceptable limit recommended in the European Union and Australian drinking water guideline, respectively. The major threat is represented by arsenic, reaching 80 µg/L. An arsenic concentration of about 50 mg/kg was reported for the Angren coal (Dill et al., 2008), and As-bearing pyrite is hosted in the kaolinized rocks of the kaolin-bearing coal deposits at Angren (Dill et al., 2008). Certainly, these deposits represent a viable As source. It is worth noting that a groundwater sample (AN_03) collected in a water hole, about 1.3 km South-West with respect to the open-pit coal mine pond, was characterized by a signature for Mo, As, and Sb. This suggests that the pond water, contaminated by coal ash and/or the composite coal-kaolin deposits, may potentially threaten groundwater through infiltration.

### Water quality for irrigation and drinking purposes

A high salinity is one of the main problems of the river water in the Syrdarya basin, which has caused many adverse effects on local people and on the ecological environment (Zhang et al., 2019 and reference therein). The Akhangaran and, in particular, the Chirchik rivers contribute significantly to the annual runoff to the upper Syrdarya, and their water quality may influence the salinization trend. Four indices were applied to evaluate the Chirchik and Akhangaran water quality in terms of irrigation suitability, considering that the agricultural area is vulnerable to soil salinization (Isaev et al., 2020), as also indicated by this study: the sodium adsorption ratio (SAR), the sodium percentage (Na%), Kelly’s index (KI), and the total hardness (TH) (Satybaldiyev et al., 2023).

**Fig. 8:**
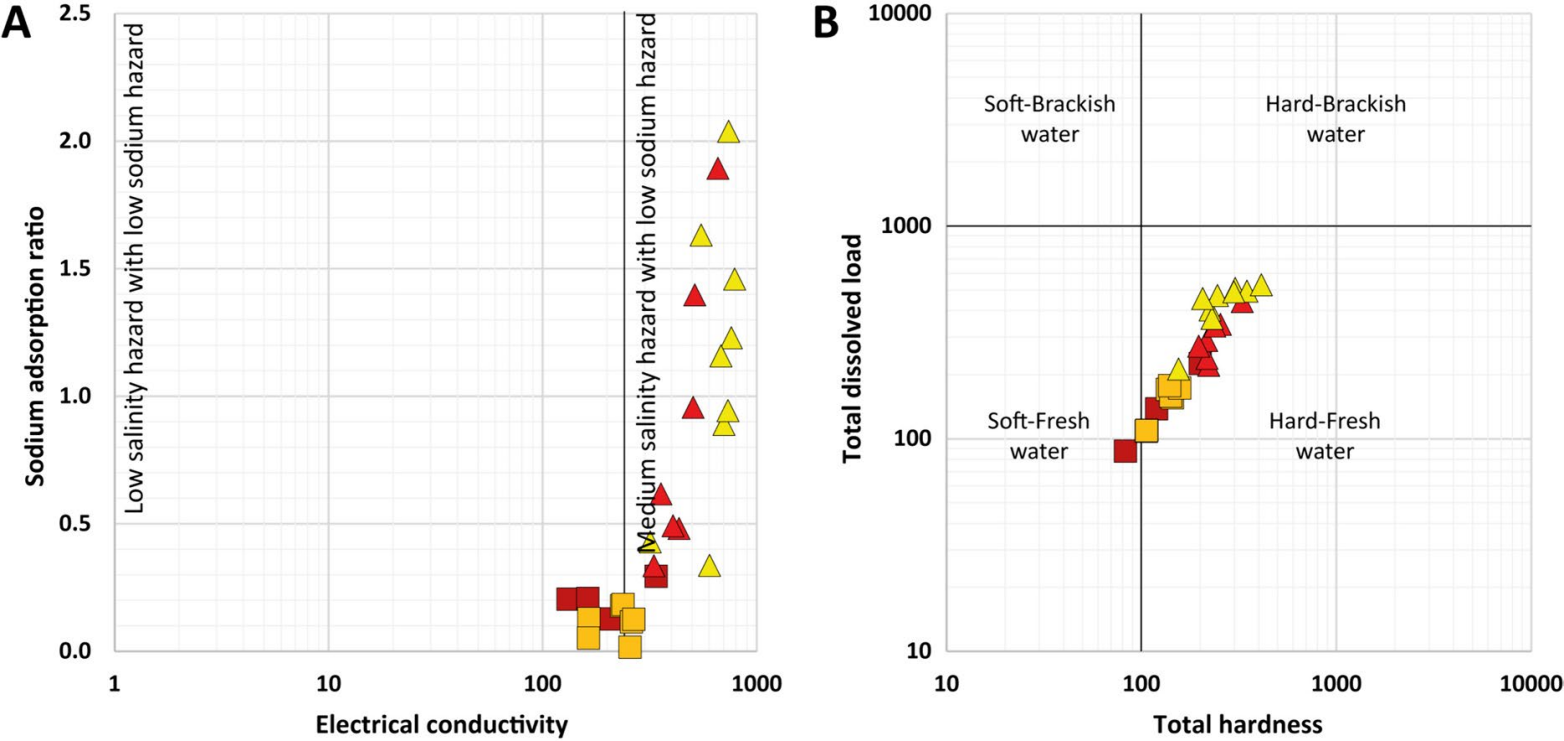
Water quality diagrams: (**A**) sodium adsorption ratio vs. electrical conductivity; (**B**) total dissolved load vs. total hardness

SAR represents the relative activity of Na^+^ in soil exchange reactions and is used with EC to evaluate the degree of alkalization of irrigation water (Fig. 8A). Waters upstream the Akhangaran and Chirchik rivers have low salinity and no sodium hazard, while water samples in the lowland area show medium to high salinity hazard, but with a low sodium hazard. Indeed, on the basis of the Na% parameter, approximately 64% of waters are classified as excellent for irrigation, and 36% are from good to permissible. On the contrary, the SAR index indicates a high salinity hazard. When classifying the suitability for irrigation on the basis of the KI index, a value larger than 1 means that there is too much sodium for irrigation, and a value less than 1 means that the water is suitable for irrigation. Despite an evident enrichment in Na^+^ in the downstream area, all the water samples investigated are suitable for irrigation. Regarding TH, all samples can be classified as hard-fresh water, except for AN_02, which is classified as soft-fresh water (Fig. 8B).

According to the US Department of Agriculture (Richards, 1968), the suitability of irrigation water can be based also on the total dissolved solids (TDS) and classified as excellent (TDS < 150 mg/L), good (TDS in the range 150–500 mg/L), doubtful (500–1500 mg/L), or unsuitable (> 1500 mg/L). Although the aforementioned indices on irrigation quality indicate that most of the sampled waters would be suitable for irrigation, on the basis of the TDS parameter, 40% of the water samples would have doubtful suitability for irrigation.

The drinking spring waters CH_01 and CH_07 meet the concentration threshold for inorganic contaminants imposed by WHO guidelines, EU, and USEPA standards, and the permissible concentration accepted by the Republic of Uzbekistan (GOST 4152–81, 2006). However, spring CH_07, in the Charvak urban settlement, is characterized by a significant NO_3_^−^ content (NO_3_^−−^ = 26.94 mg/L). Although below the Uzbekistan guideline (NO_3_^−−^ = 45 mg/L), nitrate indicates human sources; the consumption of this water can be harmful, especially for babies, and quality monitoring is needed.

## Conclusions

This study investigated the geochemistry of surface water and groundwater in the Chirchik–Akhangaran basin (Tashkent province, Uzbekistan) in order to identify the geogenic and anthropogenic contribution on the dissolved load, with implications for water quality and management plans. The major ion chemistry indicated that most waters belong to the Ca-HCO_3_ type in both the Akhangaran and Chirchik upstream catchments, despite the dominance of silicate rock outcrops. This is attributable to the role of secondary carbonates that form during the silicate weathering process, besides dissolution of carbonate rocks which are locally exposed. Waters in lowland areas differed in composition from the mountain catchments, showing a spatial pattern for some major ions. In particular, an increase in Na^+^, K^+^, SO_4_^2−^, Cl^−^, and NO_3_^−^ was observed downflow, likely reflecting the release of these ions from water-soluble salts in lands impacted by agricultural activities and livestock farming. Common water quality indices for irrigation indicated that most waters are suitable for use in irrigation; however, from the TDS content analysis, about 40% of the studied samples resulted to be in a critical state. The potentially toxic element concentration was generally lower than the thresholds established for water quality by different regulations. However, although present in low amounts, the concentration of some trace elements was higher than the averaged values reported for river waters worldwide. In addition, exceedingly high As content (besides Al, B, Ba, Mo, Sb) was measured in the Ca-SO_4_-type surface water collected in a pond within the fly-ash deposits from the Angren and Novo-Angren coal-fired power plants. Such contaminants may reach groundwater if poorly attenuated during vertical infiltration, a signature observed in the groundwater collected South-West with respect to the open-pit coal mine. Spring waters intended for human consumption were within guidelines for inorganic pollutants; however, efforts are needed to monitor the drinking water quality in a spring/fountain in the Charvak urban area, frequently used by local people.

## Data Availability

No datasets were generated or analysed during the current study.
